# The remarkable journey of one female individual with ornithine transcarbamylase deficiency diagnosed post‐mortem

**DOI:** 10.1002/jmd2.12361

**Published:** 2023-01-29

**Authors:** RaeLynn Forsyth, Ryan H. Peretz, Angela Dempsey, Jacquelyn Britton, Lisa Kratz, Ada Hamosh, Hilary Vernon, Mark L. Batshaw, David Valle

**Affiliations:** ^1^ Department of Genetic Medicine Johns Hopkins University School of Medicine Baltimore Maryland USA; ^2^ National Human Genome Research Institute National Institutes of Health Bethesda Maryland USA; ^3^ Biochemical Genetics Laboratory Kennedy Krieger Institute Baltimore Maryland USA; ^4^ Center for Genetic Medicine Research Children's National Hospital Washington DC USA

**Keywords:** CPS1, hypothermia, intellectual disability, ornithine transcarbamylase deficiency, OTC, severe SARS‐CoV‐2 infection

## Abstract

Urea cycle disorders (UCDs) comprise a group of inborn errors of metabolism with impaired ammonia clearance and an incidence of ~1:35 000 individuals. First described in the 1970s, the diagnosis and management of these disorders has evolved dramatically. We report on a 59‐year‐old woman with a UCD who contributed to advances in the understanding and treatment of this group of disorders. This individual was diagnosed with carbamoyl phosphate synthetase 1 deficiency based on a biochemical assay under a research context predating genetic sequencing, treated longitudinally as having this metabolic disorder, and was among the first participants to trial UCD pharmaceutical therapies. She ultimately succumbed to a SARS‐CoV‐2 infection while maintaining unexpectedly normal ammonium levels. Postmortem genetic testing revealed ornithine transcarbamylase deficiency. This individual's contributions to the field of UCDs is discussed herein.

**Synopsis**

The diagnosis and treatment for urea cycle disorders has evolved as evidenced by this 59‐year‐old woman with a urea cycle disorder who was initially diagnosed as having CPS1 deficiency and ultimately found to have OTC deficiency post‐mortem.

## 1 INTRODUCTION

**FIGURE 1:**
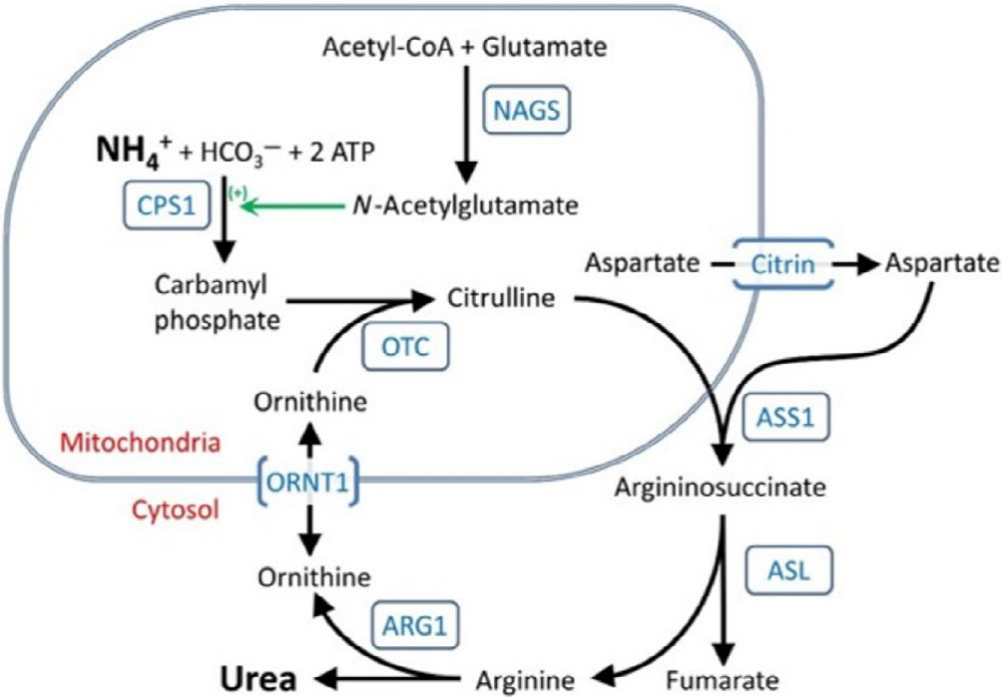
The urea cycle. *Source*: From *Urea Cycle Disorders Overview. Genereviews®*. In: Adam MP, Ardinger HH, Pagon RA, et al., eds. University of Washington, Seattle; 1993–2021. ©1993–2022 University of Washington.

Urea cycle disorders (UCDs) result from loss of function mutations in genes that encode any of the six enzymes or two amino acid transporters of the urea cycle (Figure 1) with the most common being X‐linked ornithine transcarbamylase (OTC) deficiency.^1^, ^2^, ^3^ Features vary depending on the severity of the specific genetic sequence variants though the hallmark of UCDs is acute, episodic, life‐threatening hyperammonemia.^4^ Not all UCDs, including OTC deficiency in most states, are detected by newborn screening, so physician awareness must remain high for patients of all ages presenting with symptoms suggesting a UCD.^5^ Whereas enzymatic testing on liver biopsy samples was used historically, molecular genetic testing is now widely available to confirm a diagnosis. Importantly, the activity of urea cycle enzymes varies with nutritional state, and enzymatic testing for OTC deficiency in females is complicated by X‐chromosome inactivation.^6^ An allopurinol challenge test is a useful alternative to confirm heterozygosity for OTC deficiency in females, though this test should be interpreted with caution.^7^ Currently, treatment of patients with UCDs largely consists of restriction of dietary protein and promotion of alternate pathways of waste nitrogen excretion through use of ammonia scavengers and, if necessary, hemodialysis to manage acute, unremitting hyperammonemia.^8^, ^9^ Even with these efficacious therapies, patients with UCDs are still at risk for life‐threatening hyperammonemic episodes, which prompt many to receive liver transplants.^10^ We report a female patient who was instrumental in the understanding of the diagnosis, treatment, and management of UCDs.

## 2 CASE REPORT

**FIGURE 2:**
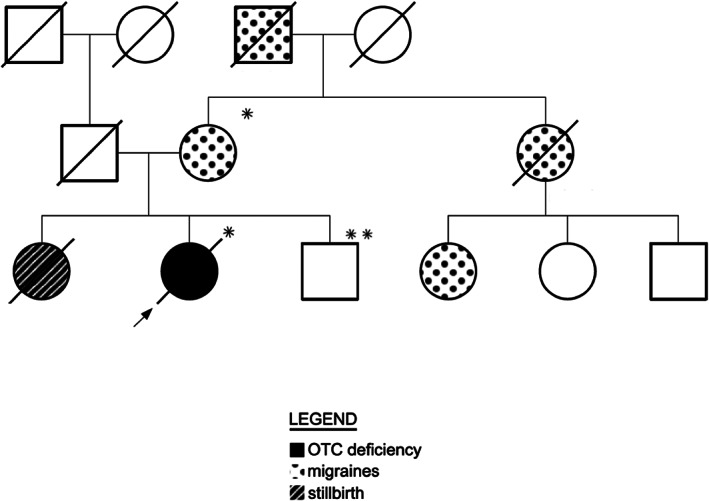
Family history. * *OTC* c.78‐1G>T variant. ** Normal *OTC* sequence.

This patient was born full term in 1961 after an uncomplicated pregnancy and delivery. Family history was remarkable for migraines on the maternal side, including the mother and several of her relatives, a stillborn sister, and a healthy brother (Figure 2). Shortly after birth, the patient began to have acute neurological episodes. At 3½ weeks of age, she had vomiting and lethargy while feeding on a milk‐based formula. These symptoms improved with administration of clear fluids, and she remained asymptomatic with normal growth and development until she had a similar presentation with postprandial vomiting at 13 months of age. No chronic neurological sequelae were obvious after these two episodes, but at 2½ years of age, she had transient left hemiparesis and was diagnosed with severe intellectual disability. At 3½ years of age, she had another episode of vomiting and became comatose but improved after 36 h of intravenous fluids. During early childhood, she continued to experience intermittent hemiparesis with hyperreflexia and spasticity, akinetic seizures (confirmed on electroencephalogram), and episodic vomiting and lethargy that were often precipitated by high protein meals. Instinctively, she self‐restricted protein from her diet.

Evaluation in her early childhood years revealed cortical atrophy and hydrocephalus, yet laboratory testing showed normal liver function without acidosis, hypoglycemia, or ketosis. Plasma ammonium was not measured initially, but in 1973, when this patient was 12 years of age, she underwent further evaluation, including biochemical testing and liver biopsy to determine the cause of her symptoms. These results revealed a plasma ammonium of 200 μmol/L (reference range < 88 μmol/L) that rose to 352 μmol/L after administration of 0.5 g/kg protein challenge. Increased levels of glutamine (1070 μmol/L [reference range 550–750 μmol/L]) and alanine (920 μmol/L [reference range 200–550 μmol/L]) in plasma provided further evidence for hyperammonemia. Her urine was negative for methylmalonic acid, and orotic acid was within normal limits. The activities of three urea cycle enzymes were measured in liver tissue obtained by percutaneous needle biopsy using normal rat liver as a control. The results showed reduced CPS1 activity (<15% of control) while OTC and ASS1 activities were normal. Based on these results, she was diagnosed with CPS1 deficiency, and this diagnosis remained in place her entire life.

She was a pioneer for the various treatments for UCDs as they were developed, including nitrogen‐free essential amino acid analogues (keto acids) and ammonia scavengers (i.e., Ammonul®).^11^, ^12^ She had irreversible brain damage and epilepsy from the time of her stroke in early childhood but remained metabolically well‐controlled for most of her life without other serious comorbidities by diet and enteral ammonia scavengers. Her few hospitalizations for hyperammonemic crises, for which she required intravenous glucose‐containing fluids and Ammonul®, were caused by dietary indiscretions involving high‐protein meals and/or intercurrent infections. She endured a prolonged admission at age 48 due to weight loss and hypothyroidism related to probable depression in response to the death of her best friend in her daycare program. During that admission, a gastrostomy tube was placed, but she did not have hyperammonemia. Over the last 10 years of her life, she experienced only four hyperammonemic episodes despite having several other periods of catabolic stress that did not result in hyperammonemia. Her diet included about 0.7–0.8 g/kg/day of protein intake, half through enteral feeds and the remainder as oral intake. In December 2020, at 59 years of age, during the height of the COVID‐19 pandemic, she presented in septic shock with bradycardia and hypothermia (rectal temperature of 90.6°F) and confirmed infection with SARS‐CoV‐2. Despite 7 days of intensive care, her respiratory failure progressed, and she died. Her ammonium levels measured at least daily were all within the normal range of <32 μmol/L. She was never placed on Ammonul® but enteral ammonia scavengers were continued. Plasma amino acid levels at presentation were notable for a normal glutamine of 477 μmol/L (reference range 337–673 μmol/L) and evidence of nutritional protein deficiency with absolute or relative decreases in the levels of tyrosine (23 μmol/L [reference range 20–108 μmol/L]), phenylalanine (15 μmol/L [reference range 25–81 μmol/L]), and the branched‐chain amino acids isoleucine (10 μmol/L [reference range 21–89 μmol/L]), leucine (29 μmol/L [reference range 40–172 μmol/L]), and valine (58 μmol/L [reference range 78–326 μmol/L]).

Because of this patient's contributions to the understanding of UCDs over her lifetime, we elected to perform postmortem molecular testing to confirm her diagnosis using a panel with 15 urea cycle related genes. The result revealed a pathogenic single nucleotide variant in *OTC* c.78‐1G>T that alters the intron 1 donor splice site.^11^ This variant has not been previously reported and is not present in the ExAC nor gnomAD databases. Familial testing showed that her unaffected brother does not have this variant, but her mother is a heterozygote (Figure 2). Other family members are not available for testing. Blood samples from prior hospitalizations in which this patient had hyperammonemia were available for further testing, but no samples showed elevations of orotic acid, a specific analyte often elevated in OTC deficiency.

## 3 DISCUSSION

We report the natural history of a 59‐year‐old woman with a UCD. Her care and management paralleled the development of modern methods of UCD diagnosis and treatment, and she has been included in two previous publications describing these developments over the last 45 years.^12^, ^13^ Together with her family, she was a participant in the clinical research that led to these developments. In an initial report, she was misdiagnosed with CPS1 deficiency based on biochemical and enzyme studies. Subsequent molecular testing obtained post‐mortem revealed that her UCD was heterozygosity for OTC deficiency. Just as this patient contributed to the understanding of UCDs throughout her life, the events surrounding her death continue to promote our learning about these rare disorders.

In retrospect, the diagnosis of OTC deficiency is more explanatory than CPS1 deficiency. First, OTC deficiency is more common than CPS1 deficiency, with prevalence of 1:56 500 compared to 1:1 300 000 individuals, respectively.^3^ Second, the maternal family history of migraines is suggestive of a possible X‐linked disorder, and females who are heterozygous for pathogenic variants of *OTC* have a spectrum of symptoms related to impaired waste nitrogen excretion, including migraine headaches.^14^ Because of the variable pattern of X‐chromosome inactivation, enzyme testing is not reliable for diagnosis of OTC heterozygosity in females. This is likely the explanation for failing to make an accurate diagnosis with an assay of OTC activity in her liver biopsy. Moreover, these assays were just being developed when performed on this patient and preanalytical handling of the liver biopsy sample was likely not as accurate as required for correct CPS1 enzyme analysis. The lack of orotic acid, a characteristic marker in OTC deficiency in blood and urine samples at times of hyperammonemia, is unusual, but orotic acid can be normal even in hemizygous male individuals with OTC deficiency.^15^

If this patient had been born today, prompt diagnosis of her OTC deficiency could have been made after her first presentation and effective therapies started before there was irreversible neurological damage. Given that OTC and CPS1 deficiencies are both proximal UCDs, medical management in this patient would not have been different had the correct diagnosis been known sooner. Accurate reproductive and familial risk counseling and testing, however, considering the X‐linked inheritance pattern, would have been offered.

Although she escaped the lethal complications of her genetic disorder, she ultimately succumbed to SARS‐CoV‐2 infection despite good metabolic control throughout this illness. This patient's relatively delayed presentation and good metabolic control with infrequent hyperammonemic episodes may have also been a clue to a partial defect and suggest that her pattern of X inactivation may have been favorable. Unfortunately, we have no data addressing this possibility. Elevated plasma ammonium levels are commonly seen in patients with UCDs during times of infection, and we would expect the SARS‐CoV‐2 virus to induce a profound catabolic state.^16^ The patient presented with hypothermia, bradycardia, and hypotension, and we hypothesize that this physiological state, in combination with the expeditious initiation of intravenous fluids with 10% dextrose to maintain a high glucose infusion rate and the introduction of enteral feeds upon patient rewarming, may have halted catabolism and prevented hyperammonemia. Hypothermia preventing hyperammonemia in the context of metabolic crises has previously been shown to be safe and feasible in neonates with UCDs, but no studies have been done in adults.^17^ This patient raises the notion of utilizing therapeutic hypothermia to help prevent catabolism and hyperammonemia among individuals affected by UCDs, which should be weighed against hemodynamic risks.^18^, ^19^

## FUNDING INFORMATION

This work was supported in part by the Intramural Research Program of the National Human Genome Research Institute at the National Institutes of Health.

## CONFLICT OF INTEREST STATEMENT

The authors declare that there is no conflicts of interest.

## INFORMED CONSENT

Informed consent was obtained from the next‐of‐kin.

## Data Availability

The data that supports the findings of this study are available within the context of this article.

## References

[1] Krebs HA , Henseleit K . Untersuchungen uber die harnstoffbildung im tierkorper. Hoppe Seylers Z Physiol Chem. 1932;210:325‐332.

[2] Colombo JP . Congenital disorders of the urea cycle and ammonia detoxification. Monogr Paediatr. 1971;1:1‐150. 4946766

[3] Summar ML , Koelker S , Freedenberg D , et al. The incidence of urea cycle disorders. Mol Genet Metab. 2013;110:179‐180. 23972786 10.1016/j.ymgme.2013.07.008 PMC4364413

[4] Matsumoto S , Häberle J , Kido J , Mitsubuchi H , Endo F , Nakamura K . Urea cycle disorders‐update. J Hum Genet. 2019;64:833‐847. 31110235 10.1038/s10038-019-0614-4

[5] State Profiles | NewSTEPs . APHL 2021. Cited January 18, 2023. https://www.newsteps.org/data-resources/state-profiles

[6] Schimke RT . Differential effects of fasting and protein‐free diets on levels of urea cycle enzymes in rat liver. J Biol Chem. 1962;237:1921‐1924. 14498420

[7] Grünewald S , Fairbanks L , Genet S , et al. How reliable is the allopurinol load in detecting carriers for ornithine transcarbamylase deficiency? J Inherit Metab Dis. 2004;27:179‐186. 15159648 10.1023/B:BOLI.0000028727.77454.bd

[8] Häberle J , Boddaert N , Burlina A , et al. Suggested guidelines for the diagnosis and management of urea cycle disorders. Orphanet J Rare Dis. 2012;7:32. 22642880 10.1186/1750-1172-7-32 PMC3488504

[9] Smith W , Diaz GA , Lichter‐Konecki U , et al. Ammonia control in children ages 2 months through 5 years with urea cycle disorders: comparison of sodium phenylbutyrate and glycerol phenylbutyrate. J Pediatr. 2013;162:1228‐1234. 23324524 10.1016/j.jpeds.2012.11.084 PMC4017326

[10] Leonard JV , McKiernan PJ . The role of liver transplantation in urea cycle disorders. Mol Genet Metab. 2004;81:S74‐S78. 15050978 10.1016/j.ymgme.2003.08.027

[11] Baralle D , Baralle M . Splicing in action: assessing disease causing sequence changes. J Med Genet. 2005;42:737‐748. 16199547 10.1136/jmg.2004.029538 PMC1735933

[12] Batshaw M , Brusilow S , Walser M . Treatment of carbamyl phosphate synthetase deficiency with keto analogues of essential amino acids. N Engl J Med. 1975;292:1085‐1090. 165404 10.1056/NEJM197505222922101

[13] Enns GM , Berry SA , Berry GT , Rhead WJ , Brusilow SW , Hamosh A . Survival after treatment with phenylacetate and benzoate for urea‐cycle disorders. N Engl J Med. 2007;356:2282‐2292. 17538087 10.1056/NEJMoa066596

[14] Maestri NE , Lord C , Glynn M , Bale A , Brusilow SW . The phenotype of ostensibly healthy women who are carriers for ornithine transcarbamylase deficiency. Medicine (Baltimore). 1998;77:389‐397. 9854602

[15] Staretz‐Chacham O , Daas S , Ulanovsky I , et al. The role of orotic acid measurement in routine newborn screening for urea cycle disorders. J Inherit Metab Dis. 2021;44:606‐617. 33190319 10.1002/jimd.12331

[16] Summar ML , Mew NA . Inborn errors of metabolism with hyperammonemia: urea cycle defects and related disorders. Pediatr Clin North Am. 2018;65:231‐246. 29502911 10.1016/j.pcl.2017.11.004

[17] Lichter‐Konecki U , Nadkarni V , Moudgil A , et al. Feasibility of adjunct therapeutic hypothermia treatment for hyperammonemia and encephalopathy due to urea cycle disorders and organic acidemias. Mol Genet Metab. 2013;109(4):354‐359. 23791307 10.1016/j.ymgme.2013.05.014

[18] Vargha R , Möslinger D , Wagner O , Golej J . Venovenous hemodiafiltration and hypothermia for treatment of cerebral edema associated with hyperammonemia. Indian Pediatr. 2012;49(1):60‐62. 22318103

[19] Ninković D , Mustapić Ž , Bartoniček D , et al. The therapeutic hypothermia in treatment of hyperammonemic encephalopathy due to urea cycle disorders and organic acidemias. Klin Padiatr. 2019;231(2):74‐79. 30870873 10.1055/a-0855-4001

